# Femoral head translation in borderline and definite dysplastic hips during weight-bearing: 2D/3D image registration analysis

**DOI:** 10.1186/s40634-023-00707-8

**Published:** 2023-11-29

**Authors:** Shinichiro Sakai, Tatsuhiko Kutsuna, Kohei Kono, Tomofumi Kinoshita, Naohiko Mashima, Masaki Takao

**Affiliations:** Department of Bone and Joint Surgery, Graduate School of Medicine, Shitsukawa, Ehime University, Toon City, Ehime 791-0295 Japan

**Keywords:** Borderline, Developmental dysplasia of the hip, Dynamic joint instability, Weight-bearing

## Abstract

**Purpose:**

The aims of this study were to 1) assess femoral head translation during weight-bearing in symptomatic developmental dysplasia of the hip (DDH) and 2) compare it between borderline DDH and definite DDH.

**Methods:**

The study included four individuals with borderline DDH and nine with definite DDH, scheduled for periacetabular osteotomy. Anteroposterior X-ray images of the hip joint were obtained in the standing position, and computed tomography images of the pelvis were obtained in the supine position. Femoral head translation from the supine to a standing position was measured using 2D/3D X-ray image registration.

**Results:**

From a supine to a standing position, the femoral head translated 0.3 mm laterally, 0.5 mm anteriorly, and 0.5 mm superiorly on average. The mean femoral head translation in 3D between the supine and standing positions was 1.5 mm. The 3D femoral head translation in the borderline DDH group was significantly greater than that in the definite DDH group. In the definite DDH group, there was a significant correlation between the center edge (CE) angle and 3D femoral head translation (*ρ* = -0.78, *P* = 0.012).

**Conclusions:**

Symptomatic DDH showed femoral head translation in the anterior, lateral, and superior directions during weight-bearing. In definite DDH, the amount of femoral head translation was negatively correlated with the CE angle. The amount of 3D translation in patients with borderline DDH was larger than that in definite DDH. Dynamic joint instability during weight-bearing was observed in borderline DDH as well as definite DDH. Treatment to enhance joint stability during weight-bearing is important in both cases.

## Background

The hip joint is generally viewed as an inherently constrained joint because of the high degree of bony congruity between the femoral head and the acetabulum [6]. Recent studies focused on dynamic instability of the hip joint as a cause of hip disorders [3, 14]. Dynamic instability of the hip joint is characterized by excessive femoral head translation within the acetabulum [16], which is determined by the bone morphology and integrity and laxity of surrounding soft tissues, including the labrum, capsular-ligamentous complex, and ligamentous teres. Much previous research has debated the morphological characteristics to diagnose hip instability; however, the optimal evaluation method of dynamic hip instability remains unclear [2, 4, 17, 20, 22, 23].

Developmental dysplasia of the hip (DDH) is an abnormality of the entire hemipelvis, which involves insufficient anterior or lateral coverage of the femoral head. Stress concentration on the limited articular surface for weight-bearing and dynamic joint instability are considered significant biomechanical factors that cause joint degeneration in younger patients [9]. Stress concentrations on the articular surface often cause damage to the labrum and cartilage. This damage, together with the lack of acetabular bony coverage and inherent soft tissue laxity, can contribute to various degrees of dynamic instability of the hip joint and cause progressive osteoarthritic changes [8, 12, 19]. However, few studies have evaluated the in vivo dynamic instability in DDH during weight-bearing [1, 18].

Some young patients with borderline DDH present with groin pain; it is unclear whether arthroscopic labral repair or reorientational osteotomy should be performed due to the lack of information on the etiology of labral and chondral damage and dynamic instability in borderline DDH.

The purpose of this study was to 1) assess femoral head translation during weight-bearing in symptomatic DDH and 2) compare it between borderline DDH and definite DDH.

## Methods

### Patients and study design

In this prospective cohort study, we evaluated 13 symptomatic hips from 13 patients with DDH who underwent rotational acetabular osteotomy between April 2018 and April 2022 at our institution.

The exclusion criteria were as follows: 1) center edge (CE) angle > 25° [21], 2) history of osteotomy surgery, 3) an osteoarthritic change of more than grade 3 according to the Kallgren-Lawrence (KL) classification, and 4) poor incongruency with the hip in abduction. All patients had bilateral dysplasia of the hip and pain in one hip joint. The diagnosis of DDH was based on anteroposterior radiography with a CE angle < 25°. We defined hips with 18° ≤ CE angle < 25° as having borderline DDH and those with CE angle < 18° as having definite DDH. Four hips were classified as having borderline DDH and nine as having definite DDH.

The mean age was 30.8 (range, 17–48) years, and all participants were female individuals. The mean body mass index was 21.8 (range, 17.7–24.0) kg/m^2^. Four patients had K-L grade 0, six patients had K-L grade 1, and three patients had K-L grade 2. None of the patients underwent surgery during infancy to reduce congenital hip dislocations. The detailed patient characteristics are shown in Table 1.

**Table 1:** Patients’ demographic and morphological parameters of the definite DDH and borderline DDH groups

	**Total Mean ± SD**	**Definite DDH Mean ± SD**	**Borderline DDH Mean ± SD**	***p* value**
Number of patients	13	9	4	
Age (years)	29.8 ± 11.0	32.2 ± 10.4	24.0 ± 8.7	0.25
Sex (female/male)	13/0	9/0	3/0	
Height (cm)	156.1 ± 4.8	154.7 ± 1.5	159.5 ± 4.7	0.098
Weight (kg)	53.1 ± 5.9	52.6 ± 2.1	54.1 ± 3.1	0.69
BMI (kg/m^2^)	21.8 ± 2.2	22.0 ± 1.1	21.3 ± 3.9	0.59
CE angle (°)	8.69 ± 11.4	3.11 ± 9.0	21.25 ± 1.7	0.0025^*^
ARO (°)	25.2 ± 12.7	31.2 ± 9.5	11.5 ± 7.0	0.0035^*^
AHI (%)	58.8 ± 12.8	52.4 ± 9.7	73.0 ± 3.8	0.0021^*^
Sharp angle (°)	51.8 ± 4.71	54.1 ± 3.8	46.5 ± 2.9	0.0018^*^
VCA angle (°)	11.2 ± 16.6	3.9 ± 10.2	27.8 ± 17.2	0.0087^*^
FEAR index (°)	6.3 ± 12.8	13.0 ± 5.4	-8.75 ± 11.7	0.0006^**^
AASA (°)	40.1 ± 8.2	38.5 ± 9.2	43.7 ± 4.6	0.31
PASA (°)	92.2 ± 6.3	90.6 ± 5.2	95.7 ± 8.0	0.19
K-L Glade	Glade 0: 4	Glade 0: 1	Glade 0: 3	
	Glade 1: 6	Glade 1: 5	Glade 1: 1	
	Glade 2: 3	Glade 2: 3	Glade 2: 0	

*SD* standard deviation, *BMI* body mass index, *CE angle* center edge angle, *ARO* acetabular roof obliquity, *AHI* acetabular-head index, *VCA angle* vertical-center-anterior angle, *FEAR index* femoro-epiphyseal acetabular roof index, *AASA* anterior acetabular sector angle, *PASA* posterior acetabular sector angle, K*-L Glade* Kallgren-Lawrence Glade

^*^Significantly different between the definite DDH group and the borderline DDH group (*p* < 0.05)

^**^Significantly different between the definite DDH group and the borderline DDH group (*p* < 0.01)

### Image acquisition

Anteroposterior X-ray images of the hip joint were obtained with the patient in the standing position using an X-ray flat panel detector system (FPD, Zexira®; Toshiba, Tokyo, Japan). Using FPD, DICOM-compliant X-ray images of the hip joint were obtained, each measuring 2048 × 2048 pixels with a 0.148-mm pixel pitch.

Computed tomography (CT) images of the pelvis were obtained using a computed tomography scanner (Philips Brilliance® 64 scanner; Marconi Medical Systems, Best, Netherlands). DICOM-compliant CT images were taken under the following conditions: resolution, 512 × 512 pixels; slice thickness, 0.67 mm; and pixel size, 0.391 × 0.391 mm.

### Two-dimensional (2D)/Three-dimensional (3D) X-ray image registration

We previously developed a unique computer-assisted image-matching procedure to analyze the kinematics of natural and artificial joints by applying an image-window-based analytical method to serial unidirectional X-ray scans. The accuracy of measurements of the patellar bone of a fresh-frozen porcine knee joint yielded a root mean square error of 0.2 mm in translation and 0.2° in rotation [11].

Briefly, the CT data were converted into voxels to construct a 3D grayscale digital image. The 3D grayscale model was located in a virtual 3D space, and a computer simulation of the radiographic process was performed to generate virtual radiographic images in which the light source and projection plane parameters were identical to those in the actual FPD imaging conditions. The relative geometric relationship between the X-ray light source and projection plane (flat panel sensors) of the FPD system was determined using a coordinate-building frame. The simulated value A of a voxel at point (*x*, *y*) on the projection plane is defined as follows:

$$A\left(x,y\right)=\overset n{\underset i{\mathrm\Sigma}}a_iL_i$$

where *a*_*i*_ is the value of the property of interest (e.g., bone mineral density) per unit length of the *i*th voxel through which a virtual X-ray beam passes, *L*_*i*_ is the length of the *i*th voxel, and *n* is the number of voxels through which a virtual X-ray beam travels.

**Fig. 1:**
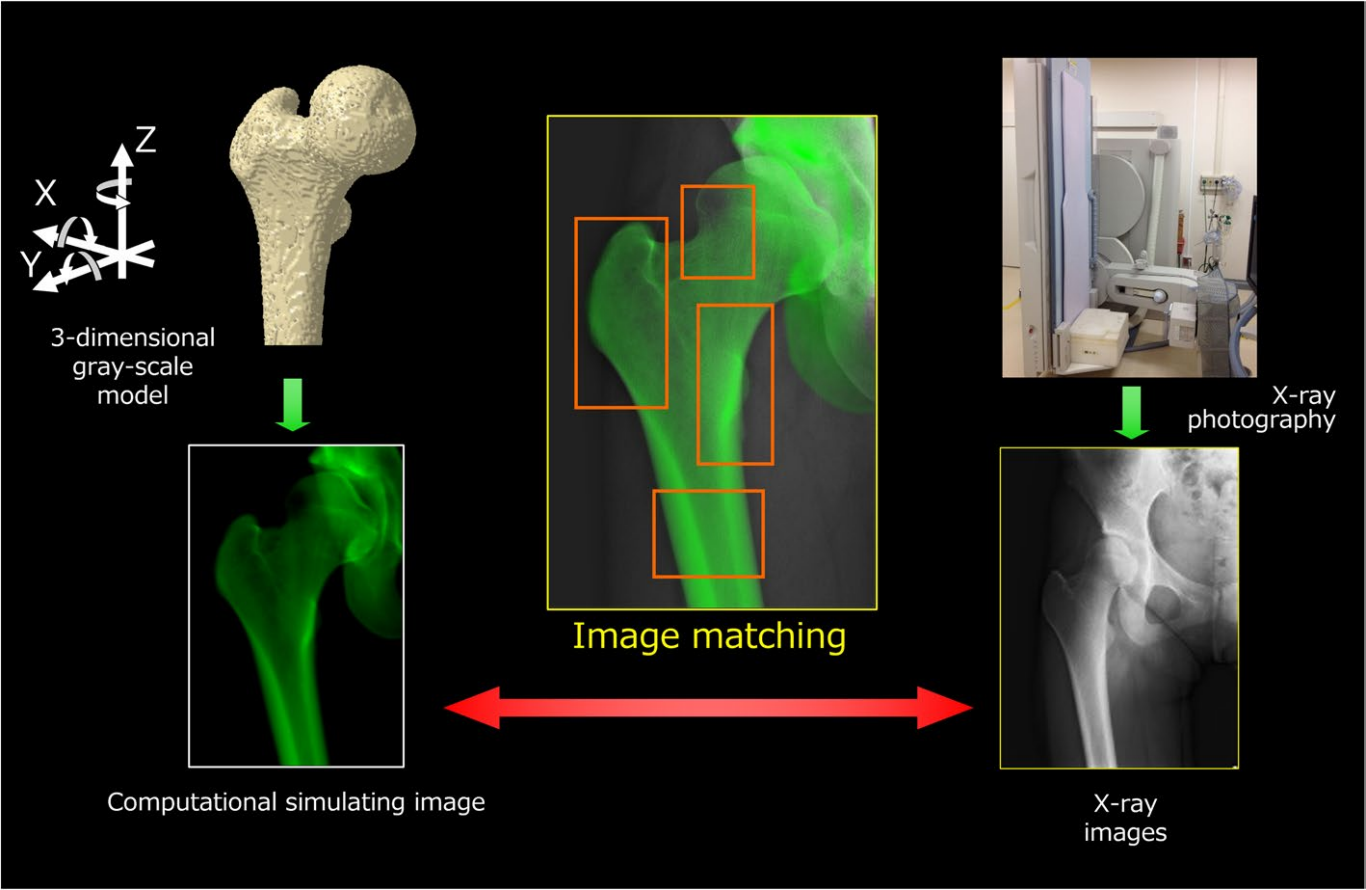
2D/3D X-ray image registration using several windows and image correlation (each x-, y-, and z-axes pointed laterally, anteriorly, and superiorly, respectively)

Virtual 2D images generated from the 3D grayscale model were then compared with the standing X-ray images acquired using the FPD. The correlations of the pixel values between the virtual and real images were used to fine-tune the 3D model (Fig. 1). Multiple small image windows spanning the bone edge were defined for image-matching analysis.

**Fig. 2:**
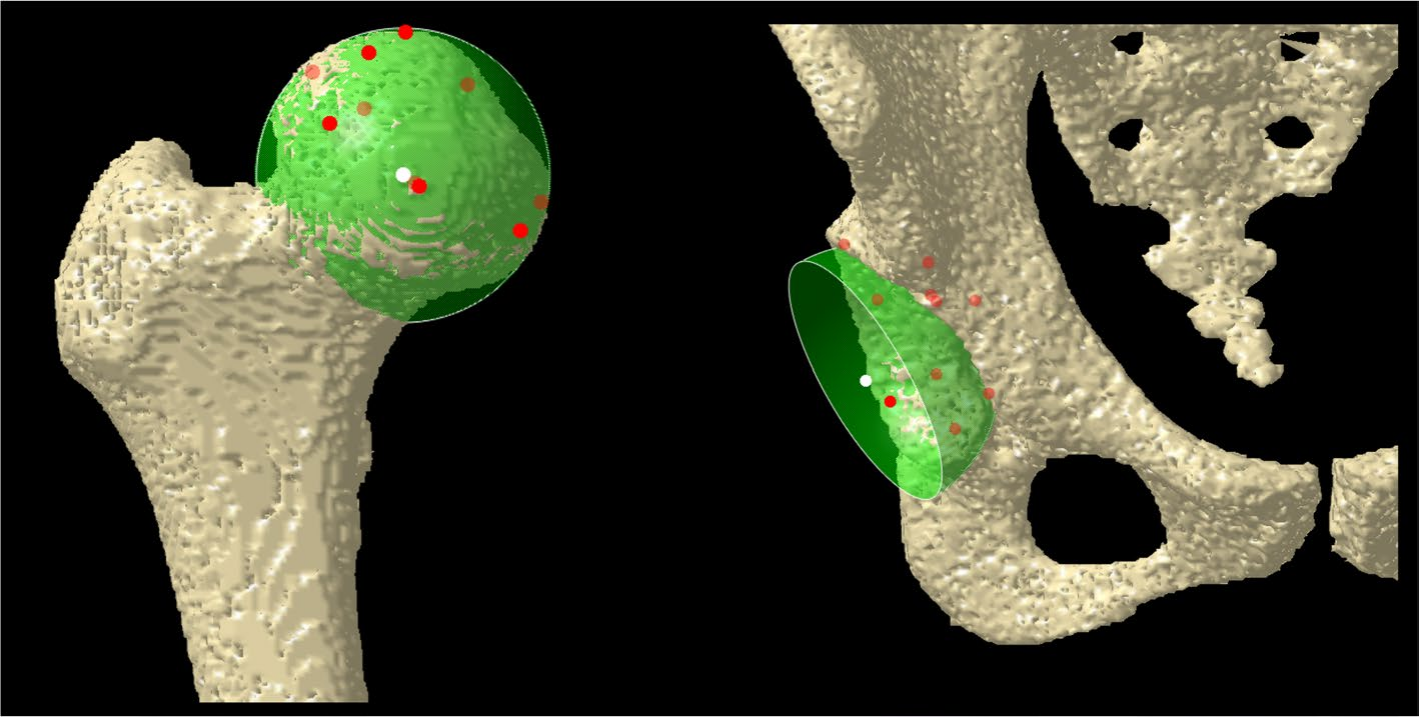
Centers of the femoral head and acetabulum are determined using the approximate sphere

**Fig. 3:**
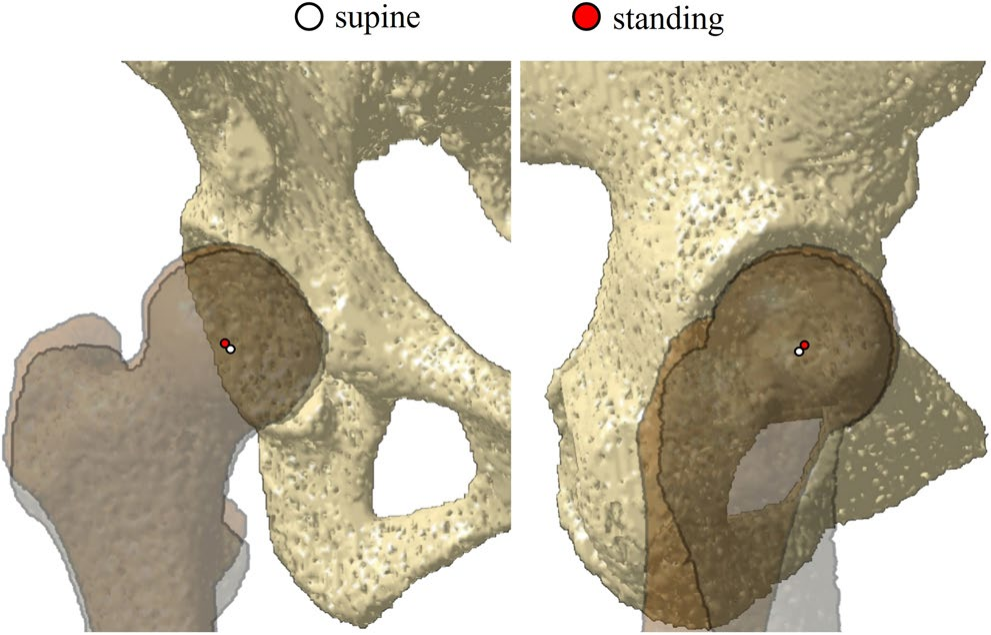
Direction and distance of femoral head center movement from supine to standing positions are measured

The centers of the femoral head and acetabulum were determined by approximating a sphere to the subchondral bone (Fig. 2). The direction and distance of movement of the femoral head center from the supine to the standing position were measured (Fig. 3). The pelvic anatomical coordinate system was used, which uses the anterior pelvic plane (APP) consisting of the bilateral anterior superior iliac spines (ASIS) and the midpoint of the bilateral pubic tuberosities as the coronal plane. Each x-, y-, and z-axes pointed laterally, anteriorly, and superiorly, respectively.

### In vitro validation study

An in vitro validation study was performed to determine the accuracy of 2D/3D image registration for this application. A porcine hip joint was prepared with the acetabulum and femur fixed using an adhesion bond. The anteroposterior X-ray view and CT images of the specimen were obtained. The 2D/3D image registration was performed separately for the pelvis and proximal femur. The femoral head translation between the CT scan and radiographic view was calculated as the error in the measurement.

### Measurement of morphological parameters

The CE angle, acetabular roof obliquity (ARO), and acetabular-head index (AHI) were measured using standing anteroposterior X-ray images and the vertical-center-anterior (VCA) angle in the false profile view. In addition, we evaluated the breakdown in Shenton’s line, Cliff sign and femoro-epiphyseal acetabular roof (FEAR) index [2, 17, 20, 22, 23]. The anterior acetabular sector angle (AASA) and posterior acetabular sector angle (PASA) were measured on the CT images.

### Postoperative evaluation

The clinical and radiographic outcomes after rotational acetabular osteotomy were evaluated over a mean follow-up period of 31 (range, 11–68) months. The Japanese Orthopaedic Association (JOA) scoring system was used for preoperative and postoperative clinical evaluation [15].

### Statistical analyses

The normality of continuous data was assessed using Levene’s test. For comparison of continuous parameters, the Student’s t-test for parametric parameters or the Mann–Whitney U test for non-parametric parameters was used. The correlation between each morphological factor and the femoral head translation in 3D was assessed using the Pearson or Spearman correlation coefficient. Statistical significance was set at *P* < 0.05. JMP® for Windows version 15.1 (SAS Institute Japan) was used for all the statistical analyses. The sample size was calculated using Power and Sample Size Calculations software version 3.1.2 (Vanderbilt University, Nashville, TN, USA). The calculation was based on a previous study, in which the femoral head translation during motion within each participant group was normally distributed with a standard deviation of 0.5 mm [1]. Consequently, if the true mean difference between the borderline DDH and definite DDH groups is 1 mm, a sample of four borderline DDH and eight definite DDH cases is required to have a statistical power of 80% to reject the null hypothesis that the population means of two groups are equal, with a type I error probability of 0.05.

### Ethics

This study was approved by the Ethics Committee of our institution (No. 1902013) and conducted in accordance with the World Medical Association Declaration of Helsinki Standard of 1964, as revised in 1983 and 2000. All patients were informed of the study in detail before they provided written informed consent for enrollment, including consent for postoperative CT imaging.

## Results

### In vivo study

From the supine to standing position, the femoral head translated 0.3 mm laterally, 0.5 mm anteriorly, and 0.5 mm superiorly on average. The mean femoral head translation in 3D between the supine and standing positions was 1.5 mm (Table 2). The 3D femoral head translation in the borderline DDH group was significantly greater than that in the definite DDH group (2.2 mm for borderline DDH vs 1.2 mm for definite DDH).

**Table 2:** Morphological parameters and amount of femoral head translation during weight-bearing in all participants

participant	Age	CE angle (°)	ARO (°)	AHI (%)	Sharp angle (°)	VCA angle (°)	Breakdown in Shenton’s line	FEAR index(°)	Cliff sign	AASA (°)	PASA (°)	X (mm)	Y (mm)	Z (mm)	3D translation (mm)
1	32	-10	42	42	58	-14	+	8	+	22	99	-0.6	1.6	0.9	1.9
2	18	-6	43	40	57	-3	+	17	+	40	84	0.3	0.3	1.6	1.7
3	48	-5	36	44	55	-1	+	21	+	33	91	0.3	-1.2	0.8	1.5
4	39	0	30	50	54	8	-	9	+	37	92	0.7	0.3	-0.4	0.9
5	32	4	33	56	51	1	+	13	+	36	95	-0.4	0.7	0.1	0.8
6	40	5	37	48	57	2	+	12	+	36	90	1.0	0.9	-0.4	1.4
7	18	12	18	63	48	6	-	8	+	43	82	0.2	0.3	-0.6	0.8
8	21	14	18	64	54	19	-	8	+	54	92	-0.5	0.1	-0.2	0.5
9	42	14	24	65	53	17	-	21	+	47	91	0.5	1.0	0.4	1.2
10	22	19	15	69	46	30	-	0	+	50	96	1.3	-1.2	1.3	2.2
11	17	21	18	73	47	3	-	-4	-	41	95	1.3	1.2	1.5	2.3
12	19	22	2	72	46	42	-	-5	+	39	106	0.2	1.9	-0.8	2.1
13	39	23	11	78	43	36	-	-26	+	45	86	-0.9	1.2	1.7	2.2

*CE angle* center edge angle, *ARO* acetabular roof obliquity, *AHI* acetabular-head index, *VCA angle* vertical-center-anterior angle, *FEAR index* femoro-epiphyseal acetabular roof index, *AASA* anterior acetabular sector angle, *PASA* posterior acetabular sector angle

**Fig. 4:**
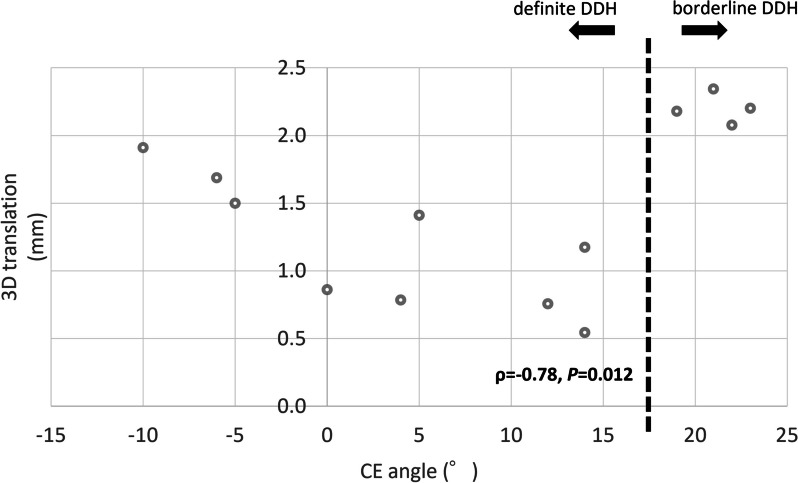
Scatter plot of the CE angle and 3D femoral head translation in borderline and definite DDH. CE, center edge; DDH, developmental dysplasia of the hip

All morphological parameters are shown in Tables 1 and 2. No morphological factors were correlated with 3D femoral head translation among whole subjects (Table 3). In contrast, in the definite DDH group, all morphological parameters, except for the PASA and FEAR index, showed a significant correlation with 3D femoral head translation (Table 3). The CE angle showed a significant correlation with 3D femoral head translation (*ρ* = -0.78, *P* = 0.012) (Fig. 4).

**Table 3:** Correlation between morphological parameters and the amount of 3D femoral head translation

Morphological parameter	All DDH (*n* = 13)		Definite DDH (*n* = 9)	
	**Spearman ρ**	***P* value**	**Spearman ρ**	***P* value**
CE angle	0.38	0.19	-0.78	0.012
ARO	-0.26	0.38	0.89	0.001
AHI	0.39	0.18	-0.81	0.007
Sharp angle	-0.32	0.27	0.81	0.007
VCA angle	0.13	0.65	-0.80	0.010
FEAR index	-0.53	0.061	0.35	0.34
AASA	-0.21	0.48	-0.68	0.042
PASA	0.37	0.20	0.13	0.73

*CE angle* center edge angle, *ARO* acetabular roof obliquity, *AHI* acetabular-head index, *VCA angle* vertical-center-anterior angle, *FEAR index* femoro-epiphyseal acetabular roof index, *AASA* anterior acetabular sector angle, *PASA* posterior acetabular sector angle

The breakdown of Shenton's line was observed in five of nine hips with definite DDH. There was no significant difference in 3D femoral head translation between groups in which the breakdown of Shenon’s line did or did not occur (1.46 ± 0.42 in the breakdown group and 1.52 ± 0.75 in the non-breakdown group, *p* = 0.88). The Cliff sign was observed in all but one hip with borderline DDH.

### In vitro validation study

The translational and rotational errors of the 2D/3D X-ray image registration were -0.10° and -0.69 mm for the X-axis direction, -0.37° and 0.21 mm for the Y-axis direction, and 0.13° and -0.09 mm for the Z-axis direction, respectively.

#### Postoperative outcomes

The JOA scores improved from 74 to 98 in the borderline DDH group and from 74 to 96 in the definite DDH group at the latest follow-up. There was no significant difference in the preoperative nor postoperative JOA scores between the groups. There were no significant differences in postoperative morphological parameters, other than ARO, between the groups (Table 4).

**Table 4:** Postoperative clinical and radiographic outcomes in the definite DDH group and the borderline DDH group

	**Total Mean ± SD**	**Definite DDH Mean ± SD**	**Borderline DDH Mean ± SD**	***p* value**
Number of patients	13	9	4	
Follow-up period (months)	31.9 ± 16.5	35 ± 18.4	25 ± 9.4	0.33
Preoperative JOA score	74.0 ± 12.5	74.2 ± 13.1	73.5 ± 13	0.92
Postoperative JOA score	96.6 ± 4.7	96.2 ± 5.4	97.5 ± 2.8	0.67
CE angle (°)	41.6 ± 7.3	39.2 ± 7.1	47.0 ± 4.8	0.074
ARO (°)	-7.2 ± 8.0	-4.3 ± 7.7	-13.7 ± 4.5	0.046*
AHI (%)	93.2 ± 6.6	91.2 ± 6.5	97.8 ± 4.4	0.09
Sharp angle (°)	36.8 ± 4.5	38.2 ± 3.6	33.5 ± 5.0	0.079
VCA angle (°)	47.6 ± 11.0	45.1 ± 3.6	53.3 ± 5.4	0.23

*JOA score* Japanese Orthopaedic Association score, *SD* standard deviation, *CE angle* center edge angle, *ARO* acetabular roof obliquity, *AHI* acetabular-head index, *VCA angle* vertical-center-anterior angle

^*^Significantly different between the definite DDH group and the borderline DDH group (*p* < 0.05)

## Discussion

We evaluated dynamic joint instability during weight-bearing in patients with symptomatic DDH, evaluated using 2D/3D image registration. Femoral head translation in the anterior, superior, and lateral directions was observed during weight-bearing. In definite DDH, the amount of femoral head translation was negatively correlated with the degree of CE angle, while that in borderline DDH was larger than that in definite DDH.

Few studies have evaluated the in vivo dynamic instability in DDH. Akiyama et al. [1] evaluated the movement of the femoral head in the acetabulum from the Patrick position to the neutral position using 3D MRI. They assessed dynamic hip joint instability under non-weight-bearing conditions in 20 dysplastic hips of 13 patients, with a CE angle less than 20^°^, and compared it with that in 40 normal hips from 22 volunteers. They observed that the femoral head translated postero-infero-medially, and the amount of the femoral head translation in 3D was 1.97 mm for DDH hips and 1.12 mm for normal hips (*P* = 0.005). The CE angle was the determinant for 3D-translation from the neutral to the Patrick position, which coincides with our results for definite DDH.

Sato et al. [18] investigated femoral head translation during weight-bearing gait using anteroposterior fluoroscopy and 3D-to-2D model-image registration in 13 patients with DDH with a CE angle of less than 20^°^. They compared the femoral head translation of the affected side with that of the contralateral healthy side. DDH hips exhibited greater swing-phase femoral translation during walking than did contralateral healthy hips. Maximum translations averaged 1.0 mm in the DDH hips and 0.5 mm in the contralateral healthy hips during the swing phase. However, there were no significant pairwise differences in the translation components during the stance phase. This might be because their study compared the affected symptomatic side and the contralateral asymptomatic healthy side of the same patient. Regression analyses showed significant correlations between maximum femoral head translation and the CE angle, AHI, and ARO, which coincides with our results for definite DDH.

Little has been reported on the dynamic instability in borderline DDH. Irie et al. [10] evaluated the distribution of subchondral bone densities in the acetabulum using CT osteoabsorptiometry to assess the physiological and biomechanical conditions of borderline-mild DDH. The high-density area did not differ proportionally between the borderline-mild DDH and the control hips; they inferred that joint instability and the consequent shear stresses may cause osteoarthritis in patients with borderline-to-mild dysplasia. In this study, higher dynamic hip instability during weight-bearing was found more commonly in borderline DDH than in definite DDH, which supports the hypothesis that the etiology of borderline DDH is instability.

Hip joint stabilization relies on acetabular bony coverage and integrity of soft tissues, including the acetabular labrum, ligamentum teres, and capsular structures [13]. Soft tissue laxity, in addition to labral and cartilage damage, can affect hip joint stabilization. Rotational acetabular osteotomy improved the symptoms of four patients with borderline DDH, suggesting that a slight variation in acetabular coverage between normal and borderline dysplastic hips could influence hip instability, but not mechanical stress concentration, in some patients. The degree of inherent joint laxity may influence susceptibility to joint instability.

This study had some limitations. First, we did not evaluate femoral head translation during weight-bearing of the normal hip joints as a control to avoid the effects of radiation exposure. Second, the definition of borderline dysplasia remains unclear. In this study, we defined hips with 18° ≤ CE angle < 25° as having borderline DDH, according to the definition by Domb et al. [5], while borderline dysplasia is often defined as 20° ≤ CE angle < 25° [7]. The controversial definitions may be because the difference between mechanical stress-dominant DDH and instability-dominant DDH is unclear. Third, the number of cases was small, despite being the minimum required number according to our power analysis. Further studies involving a larger number of DDH cases are necessary to validate our findings. Finally, the degree of femoral head translation during weight-bearing was small compared with the measurement accuracy of our computational 2D/3D image registration method (root mean square error of 0.2 mm in translation and 0.2° in rotation in porcine patellar bones [11]). The in vitro validation study using porcine hip bones, presented herein, also indicated a measurement accuracy of < 1 mm. In this study, the femoral head translation during weight-bearing in DDH ranged from 0.5 to 2.3 mm, similar values to those reported in previous studies on dynamic instability of definite DDH [1, 18]. At least, we consider that the femoral head translation during weight-bearing in symptomatic DDH could be measured using our computational 2D/3D image registration method.

## Conclusions

In symptomatic DDH, femoral head translation in the anterior, lateral, and superior directions was observed during weight-bearing. In definite DDH, the amount of femoral head translation was negatively correlated with the CE angle, whereas the translation of hips with borderline DDH was larger than that of those with definite DDH. The results of this study suggest that hip bony morphology and inherent soft tissue laxity vary among dysplastic hips, both of which contribute significantly to joint stability.

## Data Availability

All data and materials generated or analyzed during this study are included in this published article.

## Abbreviations

DDH: Developmental dysplasia of the hip
CE angle: Center edge angle
CT: Computed tomography
FPD: Flat panel detector
APP: Anterior pelvic plane
ASIS: Anterior superior iliac spines
ARO: Acetabular roof obliquity
AHI: Acetabular-head index
VCA angle: Vertical-center-anterior angle
FEAR index: Femoro-epiphyseal acetabular roof index
AASA: Anterior acetabular sector angle
PASA: Posterior acetabular sector angle
